# Diagnostic or Therapeutic Strategies for Pregnancy Complications

**DOI:** 10.3390/jcm11113144

**Published:** 2022-05-31

**Authors:** Camille Couture, Sylvie Girard

**Affiliations:** 1 Ste-Justine Hospital Research Center, Department of Microbiology, Infectiology and Immunology, Universite de Montreal, Montreal, QC H3T 1C5, Canada; camille.couture.2@umontreal.ca; 2 Department of Obstetrics & Gynecology, Department of Immunology, Mayo Clinic College of Medicine and Science, Rochester, MN 55905, USA

Pregnancy complications including preeclampsia, preterm birth, recurrent pregnancy loss, and fetal growth restriction affect over 12% of all pregnancies worldwide. However, the risks of suffering from pregnancy loss or developing pregnancy complications could be detectable at an earlier stage giving opportunity for meaningful interventions. For example, fetal sex differences, which arise very early in development due to differential gene expression from the X and Y chromosomes, could be integrated into the development of risk prediction tools for certain complications [1]. On the other hand, once a pregnancy is closer to term, uterine cervical changes can be detected using ultrasound elastography, which could in turn also improve the management of delivery [2].

Pregnancy complications have negative short- and long-term impacts on both maternal and neonatal health such as the increased risk of neurodevelopmental and cardiovascular diseases (CVD). There are currently limited ways for the early identification of such long-term health outcomes. However, the examination of the placenta and extra-placental membranes offers new opportunities, which could optimize the care given to mothers and babies who are at greater risk of neurodevelopmental and cardiovascular consequences. Women who had more severe placental lesions of maternal vascular malperfusion after preeclampsia had a three-fold increased risk of screening as high-risk for CVD compared to women without these lesions [3]. Interestingly, fetal health can also be investigated through placental pathology, where the odds of neonatal intensive care unit (NICU) admission were twice as high in pregnancies with placental pathologies in addition to placental abruption [4]. Furthermore, inflammation in the extra-placental membranes showed that the inflammatory milieu of amniotic fluid increases with chorio-deciduitis grade, but not amnionitis of these membranes [5].

Advances in the early detection of women at risk of pregnancy complications have increased in the last decade, in part due to bioinformatic improvements, making it much easier to access and analyze large amounts of data and identify novel biomarkers. Indeed, proteomic biomarkers in maternal urine have proven promising as a non-invasive way of identifying women at risk of preeclampsia and fetal growth restriction [6]. Proteomic biomarkers, this time in maternal serum samples, were also investigated in a replication study looking at the ratio of IBP4/SHBG proteins as a predictive biomarker for spontaneous preterm birth [7]. Maternal blood has additionally been used to identify markers of immune tolerance and angiogenesis, showing decreased galactin-9 and VEGF-A in patients with prior miscarriage [8].

Potential novel therapeutic targets have also been uncovered using other techniques, where for example, a polymorphism in MMP-9, correlated with high MMP-9 production, has been associated with preeclampsia [9]. Of course, identifying biomarkers and women who are at risk of pregnancy complications is not an end-all-be-all solution. Novel therapeutic strategies must also be developed in parallel to optimize the health of both mother and baby. By identifying proteins, we can then hope to target them, as has been performed by Kniotek et al., where sildenafil citrate was used to downregulate *PDE5A* expression in vitro in immune cells from patients with recurrent pregnancy loss [10]. This process could also potentially be applied to the protein SPINT1, which is reduced in preeclamptic pregnancies with co-existing fetal growth restriction, to help promote adequate blood flow and nutrient delivery to the placenta to facilitate fetal growth [11]. Similarly, another in vitro study showed that cardioprotective beta-blockers could promote the secretion of pro-angiogenic mediators in endothelial cells and mitigate inflammation, offering potential novel therapeutic strategies in preeclampsia [12].

Since we know that inflammatory pathways are dysregulated in pregnancy complications and that they can even aggravate cardiovascular disorders, they have become key targets for therapeutic strategies and drug delivery. A great example of this is IL-1, which is known to be increased in pregnancy complications and is thus a promising target in clinical settings. The therapeutic potential of blocking IL-1 in human pregnancies, as used for several inflammatory pathologies such as arthritis, was investigated in a review by Brien et al., which most importantly showed that there was no association with adverse perinatal outcomes [13]. To better understand the designs and hurdles in therapeutics for pregnancy complications, Coler et al., present possible steps to expedite drug development to better meet the growing need for effective therapeutics in preterm birth [14].

Over the past decade, numerous groups have investigated how to mitigate these effects, promote healthier pregnancies and optimize neonatal health, however, this has been difficult to translate into clinical settings due to difficulties related to early diagnostic, drug delivery, specificity, and importantly, the lack of novel therapeutic strategies. This series of fourteen articles shed light on current advances in prenatal diagnostics, knowledge gaps in the development of novel therapeutic strategies, uses of artificial intelligence to understand the placental impact of pregnancy complications, as well as recent advances in targeted drug delivery to optimize the health of both mothers and their babies.
